# Healthcare-seeking behavior for diarrhea in under-five children and associated factors in sub-Saharan Africa: a multilevel robust Poisson regression model

**DOI:** 10.3389/fpubh.2024.1441360

**Published:** 2024-10-09

**Authors:** Tsion Mulat Tebeje, Solomon Hailemariam Tesfaye, Mesfin Abebe, Binyam Tariku Seboka, Getanew Aschalew Tesfa, Beminate Lemma Seifu, Kusse Urmale Mare, Fantu Mamo Aragaw

**Affiliations:** ^1^School of Public Health, College of Health Science and Medicine, Dilla University, Dilla, Ethiopia; ^2^Department of Midwifery, College of Health Science and Medicine, Dilla University, Dilla, Ethiopia; ^3^Department of Public Health, College of Medicine and Health Sciences, Samara University, Semera, Ethiopia; ^4^Department of Nursing, College of Medicine and Health Sciences, Samara University, Semera, Ethiopia; ^5^Department of Epidemiology and Biostatistics, Institute of Public Health, College of Medicine and Health Sciences, University of Gondar, Gondar, Ethiopia

**Keywords:** diarrhea, sub-Saharan Africa, healthcare-seeking behavior, multilevel robust Poisson regression, under-five children

## Abstract

**Background:**

Diarrhea is one of the leading causes of child death in sub-Saharan Africa (SSA). Children with diarrhea who do not receive medical advice or treatment are at high risk of poor health outcomes and increased mortality. Prompt and adequate treatment is essential to mitigate these risks. However, studies that have been conducted on the factors influencing healthcare-seeking behavior (HSB) for diarrhea in under-five children in SSA are scarce. Therefore, the purpose of this research was to determine the variables related to HSB for diarrhea in children under the age of five.

**Methods:**

A secondary data analysis was conducted on the most recent data from the Demographic and Health Surveys in 35 SSA countries. The study included a total weighted sample of 51,791 children under the age of five with diarrhea. We presented the adjusted prevalence ratio and the 95% confidence interval in the multivariable multilevel robust Poisson regression analysis to show the statistical significance and strength of the association between HSB and its determinants.

**Results:**

The pooled prevalence of HSB for diarrhea in under-five children was 58.71% (95%CI: 55.39 to 62.04). Factors found to be associated with HSB included maternal age, education and working status, antenatal care visits, postnatal checkups for the child, wasting, distance to a health facility, SSA region, and country income level.

**Conclusion:**

More than 40% of under-five children with diarrhea in SSA did not receive medical advice or treatment. To improve healthcare-seeking behavior, effective health policy interventions are necessary. These include enhancing the education and employment status of mothers, promoting regular antenatal and postnatal care visits, building health facilities in close proximity, and raising awareness in the community about the importance of seeking healthcare services for malnourished children.

## Background

The region of sub-Saharan Africa has the highest rate of child mortality globally (1, 2). Although the worldwide under-five mortality rate declined from 93 per 1,000 live births in 1990 to 38 per 1,000 in 2019, the rate in SSA decreased from 184 per 1,000 in 1990 to just 74 per 1,000 in 2021 (3). Despite the significant decline in mortality, SSA still lags far behind in achieving one of the targets of the third Sustainable Development Goal (SDG), which seeks to decrease under-five mortality to at least 25 deaths per 1,000 live births in all countries by the year 2030 (4). Diarrhea is the third leading cause of death in children 1–59 months of age and the leading cause of malnutrition in under-five children. Each year, approximately 1.7 billion cases of childhood diarrheal diseases are reported worldwide, leading to the mortality of approximately 443,832 children under the age of 5 years. Diarrhea can be caused by infections of bacterial, viral, or parasitic organisms; malnutrition; contaminated water; or other causes, such as person-to-person, poor personal hygiene, and food (5).

The lack of safe drinking water and proper sanitation poses ongoing public health challenges in developing nations, primarily due to the rapid urbanization process and associated issues. These factors contribute to 94% of the cases of diarrhea reported annually worldwide (6). Approximately 20% of the population in cities in SSA relies on unsafe water sources, particularly in informal settlement areas (7).

Diarrhea hinders the proper absorption of essential nutrients such as carbohydrates, protein, potassium, zinc, and other vital substances. This, in turn, exacerbates the issue of malnutrition (8). The substantial loss of water through diarrhea can result in dehydration, electrolyte imbalances, shock, reduced mental alertness, and finally death (9). Prolonged episodes of diarrhea are additionally linked to increased morbidity and mortality rates from other diseases, negative impacts on neurological development, and growth stunting (10).

Diarrheal diseases remain a prominent public health issue and continue to be a major cause of concern. It is essential to prioritize the prompt treatment of childhood diarrheal diseases with shorter turnaround times (11, 12). The clinical treatment of acute diarrhea involves the use of low-osmolarity oral rehydration salt (ORS) and zinc supplementation, ensuring increased intake of suitable fluids and maintaining regular feeding practices (13). The proportion of effective management of diarrhea in SSA is low, ranging from 17% in Cote d’Ivoire to 38% in Niger. However, Sierra Leone stands out as an exception, with the prevalence of good practices reaching 67% (14).

Healthcare-seeking behavior (HSB) is defined as any action or inaction made by people who believe that they have a health issue or condition, with the goal of obtaining an appropriate solution (15). Mothers play a crucial role in determining the frequency and type of health institutions visited by their children, and their responsible healthcare-seeking behavior can impact the treatment outcome of childhood illnesses, including diarrhea.

The WHO’s Integrated Management of Childhood Illness Strategy (IMCIS) heavily relies on improving family and community healthcare practices for disease diagnosis and care-seeking behaviors (16, 17). Despite efforts to implement the IMCIS in developing countries, particularly in SSA, the desired targets have not been fully achieved (18). Research on mothers’ behavior of seeking healthcare for their under-five children with diarrhea revealed that the age and sex of the children, nutritional score, age and education of the mothers, wealth index, access to electronic media, severity of diarrhea, presence of healthcare facilities within a 60-min walk distance, two or more children under the age of 5 years in the family, perceived cause of acute diarrhea, and household wealth index were found to be significant predictors (19–22).

Children whose parents or caregivers fail to provide them with healthcare services when they become sick face a greater risk of experiencing poor health outcomes and increased mortality. Therefore, it is important to understand the contributing factors of healthcare service utilization (23, 24). However, evidence on the HSB in childhood diarrheal disease and its determinants in sub-Saharan Africa is scarce. Two studies conducted previously in this region were composite studies, where the outcome variable was healthcare-seeking behavior for childhood illnesses, which comprises diarrhea and acute respiratory tract infection symptoms (fever and cough) (25, 26). Another study included two outcome variables: healthcare-seeking behavior for acute respiratory tract infection and diarrhea (27). They reported odds ratios to measure the association between the dependent and independent variables, which overestimated the relationship because the prevalence was greater than 10%. Thus, a proper statistical approach that considers the hierarchical nature of DHS data and the common occurrence of the magnitude of the dependent variable, which is a multilevel robust Poisson regression model, is appropriate. This study, therefore, seeks to estimate the pooled magnitude and examine the individual- and community-level determinants of HSB for diarrhea among under-five children in 35 sub-Saharan African countries by utilizing a mixed-effect Poisson regression model with robust variance.

## Methods

### Data source, setting, and population

The data utilized in this study were obtained from the most recent standard demographic and health survey (DHS) in sub-Saharan African countries conducted since 2010. Among SSA countries, 43 have DHS reports. Of these, countries with no DHS reports after 2010, restricted data, or no publicly available data (Botswana, Cape Verde, Central Africa Republic, Equatorial Guinea, Eritrea, Eswatini, Sao Tome and Principe, and Sudan) were excluded. In the end, we included 35 SSA countries. In this multicountry study, we included countries from the four regions defined by geographical location in the SSA: Central Africa (Angola, Congo Democratic Republic, Congo, Cameroon, Gabon, and Chad), Eastern Africa (Burundi, Ethiopia, Kenya, Comoros, Madagascar, Malawi, Mozambique, Rwanda, Tanzania, Uganda, Zambia, and Zimbabwe), Western Africa (Burkina Faso, Benin, Cote d’Ivoire, Ghana, Gambia, Guinea, Liberia, Mali, Mauritania, Nigeria, Niger, Sierra Leone, Senegal, and Togo), and southern Africa (Lesotho, Namibia, and South Africa) (Table 1) (28). Each survey was conducted at the population level and was representative of its own country, making it a large-scale study with substantial sample sizes. A two-stage cluster sampling technique was used to select the respondents in all these surveys (29). The research was conducted using the *kids’* record (KR) dataset from the DHS website: www.dhsprogram.com, which is available for public use. Children under the age of 5 years who had diarrhea in SSA were the source population, and the study population included all children under the age of 5 years who had diarrhea in the 2 weeks preceding the survey. A total of 51,791 weighted samples were included in this analysis.

**Table 1:** Sample size determination of HSB for diarrhea among children aged 0–59 months in each SSA country.

Region	Country	DHS year	Healthcare-seeking behavior		Weighted frequency (%)
			Yes (%)	No (%)	
East Africa	Burundi	2016/17	1718 (60.2)	1,138 (39.8)	2,856 (5.5)
	Ethiopia	2016	537 (44.8)	661 (55.2)	1,198 (2.3)
	Kenya	2022	1,399 (59.2)	963 (40.8)	2,362 (4.6)
	Comoros	2012	262 (53.6)	227 (46.4)	489 (0.94)
	Madagascar	2021	448 (42.4)	608 (57.6)	1,056 (2.04)
	Malawi	2015/16	2,374 (66.7)	1,185 (33.3)	3,559 (6.9)
	Mozambique	2011	765 (63.9)	432 (36.1)	1,198 (2.3)
	Rwanda	2019/20	598 (53.2)	526 (46.8)	1,124 (2.2)
	Tanzania	2022	595 (66.3)	302 (33.7)	897 (1.7)
	Uganda	2016	1931 (70.6)	805 (29.4)	2,736 (5.3)
	Zambia	2018	964 (69.6)	422 (30.4)	1,386 (2.7)
	Zimbabwe	2015	406 (42.2)	556 (57.8)	961 (1.9)
Central Africa	Angola	2015/16	981 (50.3)	971 (49.7)	1952 (3.8)
	Congo Democratic Republic	2013/14	1,633 (58.3)	1,168 (41.7)	2,805 (5.4)
	Congo	2011/12	767 (55.9)	604 (44.1)	1,371 (2.7)
	Cameroon	2018	609 (56.5)	469 (43.5)	1,078 (2.1)
	Gabon	2019/21	563 (59.5)	383 (40.5)	947 (1.8)
	Chad	2014/15	1846 (50.8)	1791 (49.2)	3,654 (7.1)
West Africa	Burkina Faso	2021	1,179 (65.5)	621 (34.5)	1801 (3.5)
	Benin	2017/18	582 (44.9)	715 (55.1)	1,298 (2.5)
	Cote d’Ivoire	2021	588 (57.9)	427 (42.0)	1,015 (2.0)
	Ghana	2014	430 (69.2)	191 (30.8)	621 (1.2)
	Gambia	2019/20	853 (62.6)	510 (37.4)	1,363 (2.6)
	Guinea	2018	746 (72.8)	279 (27.2)	1,024 (2.0)
	Liberia	2019/20	513 (68.7)	234 (31.3)	747 (1.4)
	Mali	2018	903 (55.8)	716 (44.2)	1,619 (3.1)
	Mauritania	2019/21	470 (34.0)	913 (66.0)	1,384 (2.7)
	Nigeria	2018	2,645 (67.7)	1,265 (32.3)	3,910 (7.5)
	Niger	2012	1,081 (63.3)	627 (36.7)	1708 (3.3)
	Sierra Leone	2019	472 (75.9)	150 (24.1)	622 (1.2)
	Senegal	2019	330 (46.6)	378 (53.4)	708 (1.4)
	Togo	2013/14	489 (53.1)	432 (46.9)	924 (1.8)
Southern Africa	Lesotho	2014	182 (57.4)	135 (42.6)	318 (0.61)
	Namibia	2013	528 (68.1)	247 (31.9)	778 (1.5)
	South Africa	2016	221 (63.4)	127 (36.6)	349 (0.67)

### Study variables

#### Dependent variable

The dependent variable was healthcare-seeking behavior for diarrhea in children under the age of five. In the DHS, mothers of children aged 0–59 months were asked whether their children had a history of diarrhea in the 2 weeks preceding the survey. A “yes” response indicated that the child had suffered from diarrhea during that period, whereas a “no” response indicated otherwise (30). The dependent variable (HSB) was subsequently categorized on the basis of whether medical advice or treatment was sought for children with diarrhea, with a “yes” indicating that medical attention was sought and a “no” indicating that medical attention was not sought. The respondents were asked, “Where did you seek advice or treatment?” Seeking medical attention is considered when advice or treatment is sought from any public sector, private medical sector, religious or voluntary sector, or other sources, excluding treatment from traditional healers (31).

#### Independent variables

The individual-level variables included sex and age of the child, birth order, postnatal checkup of the child, preceding birth interval, birth weight, maternal age, maternal education level, marital status, ANC visits during pregnancy, working status, sex of the household head, number of children under the age of 5 years, and the wealth index. Three factors were used to assess media exposure: reading newspapers, listening to the radio, and watching television. Mothers were then classified as not having media exposure if they had no exposure to any of the above media sources and as having media exposure if they had been exposed to at least one of the three sources. The nutritional index was measured according to the Child Growth Standards proposed by the WHO. The z scores of height for age, weight for height, and weight for age were used to measure stunting, wasting, and underweight, respectively. Compared to the references, children below −3 and between −3 and − 2 standard deviations were considered severe and moderate malnutrition, respectively (30).

The community-level variables include distance to a health facility, region in SSA, residence, and income level. In accordance with the World Bank’s list of economic classifications, the income level was categorized as lower income, lower-middle income, or upper-middle income (32).

### Data management and analysis

Statistical analysis was conducted via Stata version 17.0. Initially, each country in the SSA region was assigned a code and appended to form a unified dataset. The dependent and independent variables were subsequently extracted, cleaned, coded, and weighted (to restore the representativeness of the survey). The individual weight (v005), calculated by multiplying the household weight (hv005) by the inverse of the individual response rate for women in the stratum, was selected. We divided (v005) by 1,000,000 to calculate individual sample weights (30). Descriptive statistics, including frequencies and percentages, were calculated, followed by inferential analysis. The study analyzed the pooled prevalence of HSB for diarrhea in children under the age of five in 35 SSA countries via the STATA command “metan” and displayed it in a forest plot. To reduce the heterogeneity between regions in SSA countries, a subgroup analysis was performed.

As the study was cross-sectional and the percentage of children under the age of five with diarrhea for whom healthcare was sought was 59%, this indicates that the size of the outcome variable is common. When the prevalence exceeds 10%, reporting the odds ratio may exaggerate the association between the dependent and independent variables. This makes the prevalence ratio the most suitable measure of association for this research (33). A multilevel Poisson regression model with robust variance was fitted to calculate the prevalence ratio (34). We fitted a multilevel model to account for the hierarchical structure of the DHS data, with children nested within clusters, which represent enumeration areas in each country.

The intraclass correlation coefficient (ICC) and median odds ratio (MOR) were calculated to quantify the variance between the clusters. The ICC measures the degree of heterogeneity between clusters (the proportion of total observed individual variation in HSB for diarrhea in under-five children attributable to cluster variation).


$$ICC=V_{A}/\left(V_{A}+V_{I}\right)$$

where V_A_ is the community-level variance, and V_I_ is the individual-level variance (35).

The MOR was estimated to quantify the heterogeneity or variation in HSB for childhood diarrhea between clusters in terms of the odds ratio scale and is defined as the median value of the odds ratio between the cluster with a high likelihood of HSB for diarrhea and the cluster with a lower likelihood when individuals from the two clusters were randomly selected.


$$\mathrm{MOR}=\exp\left[\sqrt{\left(2\times V_{A}\right)\times 0.6745}\right]$$

$$\approx \exp\left(0.95\sqrt{V_{A}}\right)$$

where VA represents the cluster variance (36).

In the bivariable multilevel Poisson regression analysis, variables with a *p*-value of less than 0.2 were taken into account for the multivariable analysis. We independently fitted four models for the multivariable multilevel Poisson regression analysis. Without the use of an independent variables, Model 1 (null model) was fitted to determine how differently healthcare-seeking behavior varied throughout the cluster. We adjusted Model 2 and Model 3 for individual- and community-level variables, respectively. Model 4 was the final model fitted simultaneously for the individual- and community-level variables. The aforementioned models were compared by deviance (−2Log-likelihood ratio (−2LLR)), and the best-fit model was identified as the one with the lowest deviance. Finally, the adjusted prevalence ratio (APR) was presented with its 95% confidence interval (CI) to assess the strength and statistical significance of the associations.

## Results

### Background characteristics of the study participants

The analysis involved a weighted sample of 51,791 under-five children with diarrhea in sub-Saharan African countries. The majority of the mothers were between 20 and 35 years old (39,277, 75.8%), with a mean of 28.2 years (SD = 6.9). More than one-third of the mothers had no formal education (18,944, 36.6%), and 18,436 (35.6%) had primary education. A larger proportion of mothers reported that they were currently married (34,808, 67.2%), and 22,703 mothers (43.8%) reported that they had no access to media. Among the participants, 23,997 (46.3%) came from poor households (1st and 2nd wealth quintiles), while 17,464 (33.7%) were from rich households (4th and 5th wealth quintiles). Approximately 35,433 participants (68.4%) were living in rural areas, and 20,042 (42.0%) responded that distance to a healthcare facility was a problem. Nearly three-fourths of the participants (38,564, 74.5%) lived in eastern and western Africa, whereas only 1,441 (2.8%) lived in southern Africa. The study also revealed that the majority of the respondents (62.1%) were from lower-income SSA countries (Table 2).

**Table 2:** Distribution of the characteristics of mothers and children by HSB for diarrhea among children aged 0–59 months in SSA.

Variable		Healthcare-seeking behavior		Weighted frequency (%)
		Yes (%)	No (%)	
Maternal age	15–19	2,370 (57.0)	1786 (43.0)	4,156 (8.0)
	20–35	23,468 (59.7)	15,809 (40.3)	39,277 (75.8)
	36–49	4,770 (57.1)	3,588 (42.9)	8,358 (16.1)
Educational level of the mother	No education	10,490 (55.4)	8,454 (44.6)	18,944 (36.6)
	Primary	11,249 (61.0)	7,187 (38.9)	18,436 (35.6)
	Secondary and above	8,870 (61.6)	5,541 (38.4)	14,411 (27.8)
Working status of the mother	Not working	11,319 (56.4)	8,742 (43.6)	20,062 (38.8)
	Working	19,263 (60.8)	12,407 (39.2)	31,670 (61.2)
Marital status	Single	10,038 (59.1)	6,945 (40.9)	16,983 (32.8)
	Married	20,571 (59.1)	14,237 (40.9)	34,808 (67.2)
Wealth index	Poor	13,728 (57.2)	10,270 (42.8)	23,997 (46.3)
	Middle	6,201 (60.0)	4,129 (40.0)	10,330 (20.0)
	Rich	10,681 (61.2)	6,783 (38.8)	17,464 (33.7)
Media exposure	No	13,047 (57.5)	9,656 (42.5)	22,703 (43.8)
	Yes	17,561 (60.4)	11,525 (39.6)	29,086 (56.2)
Sex of household head	Male	24,261 (59.1)	16,773 (40.9)	41,034 (79.2)
	Female	6,348 (59.0)	4,409 (41.0)	10,757 (20.8)
Antenatal care visit	No	2,197 (47.3)	2,444 (52.7)	4,641 (10.9)
	1–3 times	8,044 (58.0)	5,836 (42.0)	13,880 (32.8)
	4 or more times	15,140 (63.6)	8,683 (36.4)	23,823 (56.3)
Age of the child	Neonate	126 (31.6)	274 (68.4)	400 (0.77)
	Post-neonate	7,135 (57.3)	5,314 (42.7)	12,449 (24.0)
	Child	23,348 (59.9)	15,594 (40.1)	38,942 (75.2)
Sex of the child	Female	14,439 (58.7)	10,160 (41.3)	24,599 (47.5)
	Male	16,170 (59.5)	11,022 (40.5)	27,192 (52.5)
Preceding birth interval in months	< 24	4,314 (57.4)	3,200 (42.6)	7,515 (14.5)
	24 and above	19,105 (58.9)	13,332 (41.1)	32,437 (62.6)
	No previous birth	7,190 (60.7)	4,650 (39.3)	11,839 (22.9)
Birth order	Above 3	12,665 (57.9)	9,216 (42.1)	21,881 (42.2)
	≤ 3	17,944 (60.0)	11,966 (40.0)	29,911 (57.8)
Birth weight	Large	10,941 (61.5)	6,860 (38.5)	17,801 (36.0)
	Average	12,699 (58.8)	8,910 (41.2)	21,609 (43.7)
	Small	5,286 (56.0)	4,160 (44.0)	9,446 (19.1)
	Unknown	311 (51.0)	297 (49.0)	608 (1.2)
Postnatal checkup within 2 months	Yes	9,458 (63.0)	5,565 (37.0)	15,023 (36.3)
	No	15,265 (58.0)	11,061 (42.0)	26,326 (63.7)
Currently breastfeeding	No	14,458 (59.2)	9,967 (40.8)	24,425 (48.5)
	Yes	15,292 (59.1)	10,602 (40.9)	25,894 (51.5)
Stunting	Normal	11,192 (56.3)	8,690 (43.7)	19,882 (65.8)
	Moderate	3,402 (56.0)	2,670 (44.0)	6,072 (20.1)
	Sever	2,391 (55.9)	1883 (44.1)	4,274 (14.1)
Wasting	Normal	15,443 (55.9)	12,203 (44.1)	27,646 (91.2)
	Moderate	1,153 (59.4)	788 (40.6)	1941 (6.4)
	Sever	434 (59.9)	290 (40.1)	724 (2.4)
Underweight	Normal	13,532 (56.3)	10,501 (43.7)	24,033 (79.1)
	Moderate	2,434 (55.8)	1930 (44.2)	4,364 (14.4)
	Sever	1,121 (56.0)	881 (44.0)	2002 (6.5)
Distance to health facility	Big problem	11,463 (57.2)	8,579 (42.8)	20,042 (42.0)
	Not big problem	16,962 (61.3)	10,691 (38.7)	27,652 (58.0)
Residence	Urban	9,757 (59.6)	6,601 (40.4)	16,358 (31.6)
	Rural	20,852 (58.8)	14,581 (41.2)	35,433 (68.4)
Region in SSA	Central Africa	6,399 (54.3)	5,387 (45.7)	11,786 (22.8)
	East Africa	11,997 (60.5)	7,827 (39.5)	19,824 (38.3)
	Southern Africa	931 (64.6)	510 (35.4)	1,441 (2.8)
	West Africa	11,282 (60.2)	7,458 (39.8)	18,740 (36.2)
Country income level in SSA	Lower	19,263 (59.9)	12,902 (40.1)	32,166 (62.1)
	Lower-middle	10,034 (57.1)	7,522 (42.9)	17,555 (33.9)
	Upper-middle	1,312 (63.4)	21,182 (36.6)	2070 (4.00)

More than half of the children (27,192, 52.5%) were male, and 24,599 (47.5%) were female, with a mean age of 23.4 months (SD = 14.8). A quarter of the children were neonates and post-neonates, and the majority (26,326, 63.7%) did not have a postnatal checkup within 2 months after delivery. Approximately 7,515 (14.5%) of the children had a preceding birth interval of less than 2 years, whereas the majority, 32,437 (62.6%), had a birth interval of more than 2 years. In terms of nutritional status, 4,274 (14.1%), 724 (2.4%), and 2,002 (6.5%) children were severely stunted, severely wasted, and severely underweight, respectively (Table 2).

### Pooled prevalence of HSB for diarrhea among children aged 0–59 months in SSA

**Figure 1:**
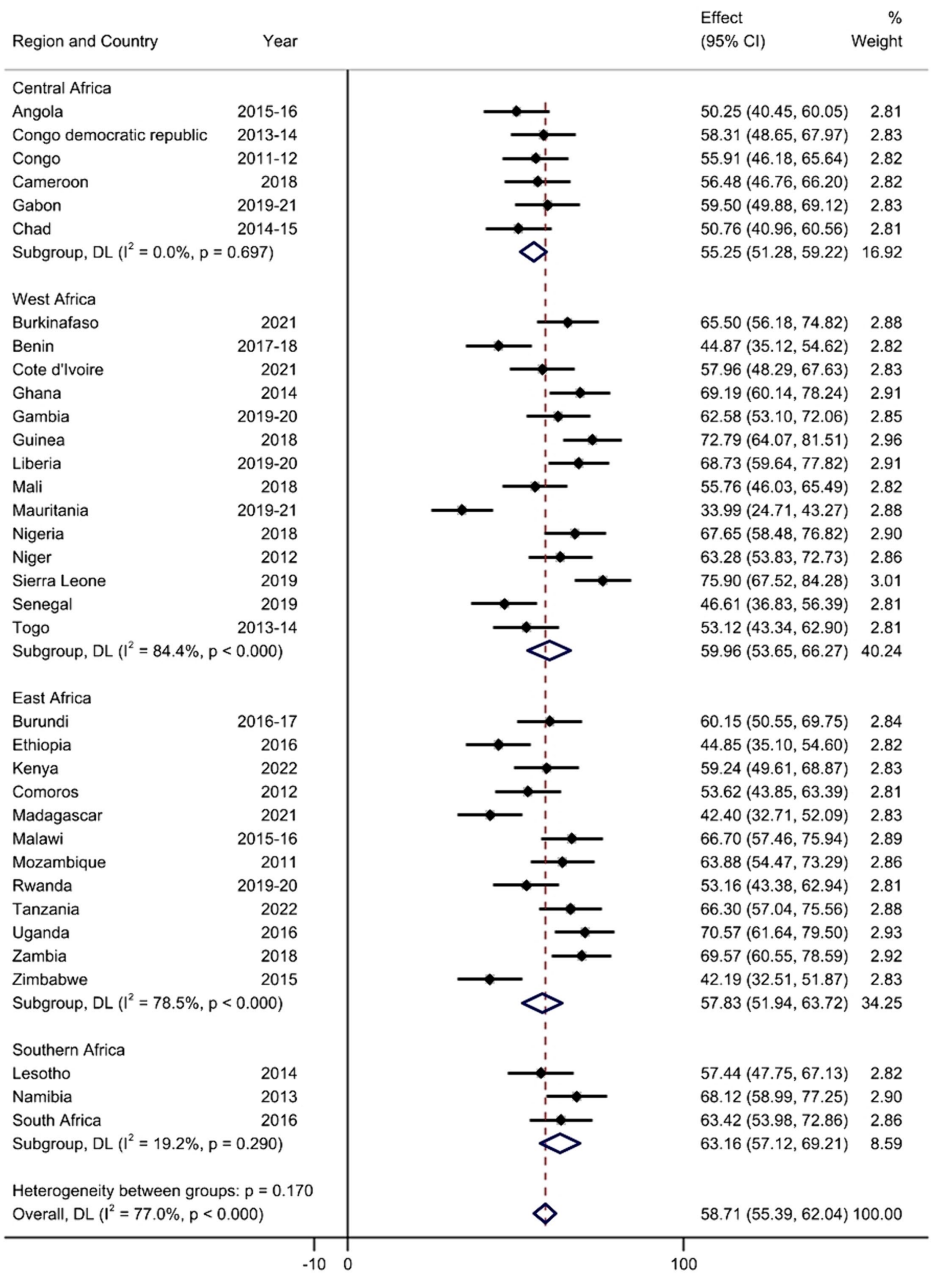
Forest plot of the pooled prevalence of HSB for diarrhea among children aged 0–59 months in SSA.

The pooled prevalence of HSB for diarrhea among under-five children in SSA was 58.71% (95% CI: 55.39, 62.04). The lowest and highest pooled prevalence rates were observed in Mauritania, with a prevalence of 33.99% (95% CI: 24.71, 43.27), and Sierra Leone, with a prevalence of 75.9% (95% CI: 67.52, 84.28), respectively. According to the subgroup analysis of regions in SSA, the highest magnitude of HSB was in southern Africa at 63.16% (95% CI: 57.12, 69.21), and the lowest was in central Africa at 55.25% (95% CI: 51.28, 59.22) (Figure 1).

### Associated factors of HSB for diarrhea among children aged 0–59 months in SSA

In the null model, the ICC value was 0.149, indicating that 14.9% of the total variability for HSB was due to differences between clusters, whereas the remaining 85.1% was attributed to individual differences. In addition, according to the MOR, if two children under the age of 5 years with diarrhea are randomly chosen from separate clusters and a child from a cluster with a lower likelihood of HSB is moved to a cluster with a higher likelihood of HSB, his/her chances of receiving healthcare for diarrhea may increase by 2.38 times. After the multilevel robust Poisson regression model was fitted, the models were compared based on their deviance values. The final model, with the lowest deviance value (deviance = 36505.52), was chosen as the best-fitting model.

In the multivariable multilevel Poisson regression model (model IV) with robust variance, the mother’s age, maternal educational status, maternal working status, ANC visits, postnatal checkups for the baby, and wasting were the individual-level determinants associated with HSB for under-five children with diarrhea. Distance to health facilities, SSA region, and country income level were the community-level determinants associated with HSB for diarrhea in under-five children.

Compared to mothers who had no formal education, those who had completed primary, and secondary or higher education had 1.05 times (APR = 1.05, 95% CI: 1.02, 1.08) and 1.05 times (APR = 1.05, 95% CI: 1.01, 1.09) greater prevalence of HSB for diarrhea in under-five children. Compared to mothers aged 15–19 years, those aged 20–35 years had a 1.06-fold (APR = 1.06, 95% CI: 1.01, 1.12) increased prevalence of HSB for their under-five children with diarrhea. Compared to their counterparts, working mothers had a 1.06 times (APR = 1.06, 95% CI: 1.03, 1.09) greater prevalence of HSB for diarrhea in children under the age of 5 years. Compared to mothers without ANC visits, those who had 1–3, or 4 or more ANC visits during the index pregnancy had a 1.22 (APR = 1.22, 95% CI: 1.16, 1.29) and 1.34 (APR = 1.34, 95% CI: 1.27, 1.41) times greater prevalence of HSB for their under-five children with diarrhea. The prevalence of receiving healthcare for diarrhea in under-five children who had postnatal checkups at a health facility within 2 months after delivery was 1.05 times (APR = 1.05, 95% CI: 1.03, 1.08) greater than that in their counterparts. Compared to normal children, under-five children who had moderate or severe wasting had a 1.08 (APR = 1.08, 95% CI: 1.03, 1.13) and 1.13 (APR = 1.13, 95% CI: 1.06, 1.21) times greater prevalence of receiving HSB for diarrhea, respectively.

Compared to their counterparts, mothers who did not experience significant difficulty with distance to health facilities had a 1.05-fold (APR = 1.05, 95% CI: 1.02, 1.07) increased prevalence of HSB for diarrhea in their under-five children. The prevalence of HSB for diarrhea in under-five children was 5% lower (APR = 0.95, 95% CI: 0.92, 0.98) among those from lower-middle-income SSA countries than those from lower-income SSA countries. With respect to the region in SSA, East Africa had a 1.08-fold (APR = 1.08, 95% CI: 1.04, 1.13) greater prevalence of HSB for diarrhea among children under the age of 5 years than Central Africa (Table 3).

**Table 3:** Multivariable multilevel Poisson regression model with robust variance analysis of factors associated with HSB for diarrhea among children aged 0–59 months in SSA.

Variable	Null model	Model 2	Model 3	Model 4
		PR (95%CI)	PR (95%CI)	PR (95%CI)
Sex of the child				
Male		1		1
Female		0.99 (0.97, 1.01)		0.99 (0.98, 1.01)
Birth order				
≤ 3		1		1
Above 3		1.00 (0.98, 1.03)		1.02 (0.99, 1.04)
Postnatal checkup within 2 months				
No		1		1
Yes		1.05 (1.03, 1.07)		1.05 (1.03, 1.08)*
Preceding birth interval in months				
No previous birth		1		1
< 24		0.98 (0.94, 1.02)		0.98 (0.94, 1.03)
24 and above		0.98 (0.95, 1.01)		0.99 (0.96, 1.02)
Maternal age				
15–19		1		1
20–35		1.08 (1.03, 1.13)		1.06 (1.01, 1.12)*
36–49		1.03 (0.98, 1.09)		1.01 (0.95, 1.07)
Educational level of the mother				
No education		1		1
Primary		1.07 (1.04, 1.10)		1.05 (1.02, 1.08)*
Secondary and above		1.05 (1.02, 1.09)		1.05 (1.01, 1.09)*
Marital status				
Single		1		1
Married		1.04 (1.01, 1.06)		1.02 (0.99, 1.06)
Antenatal care visits				
No		1		1
1–3 times		1.26 (1.19, 1.32)		1.22 (1.16, 1.29)*
4 or more times		1.38 (1.31, 1.45)		1.34 (1.27, 1.41)*
Working status of the mother				
Not working		1		1
Working		1.06 (1.03, 1.08)		1.06 (1.03, 1.09)*
Wealth index				
Poor		1		1
Middle		1.01 (0.98, 1.05)		1.02 (0.98, 1.05)
Rich		1.02 (0.99, 1.05)		1.01 (0.97, 1.04)
Media exposure				
No		1		1
Yes		1.01 (0.98, 1.03)		1.01 (0.99, 1.04)
Wasting				
Normal		1		1
Moderate		1.09 (1.05, 1.14)		1.08 (1.03, 1.13)*
Severe		1.11 (1.04, 1.18)		1.13 (1.06, 1.21)*
Distance to health facility				
Big problem		1	1	1
Not big problem			1.06 (1.04, 1.08)	1.05 (1.02, 1.07)*
Residence				
Urban		1	1	1
Rural			0.98 (0.96, 1.00)	1.01 (0.97, 1.04)
Region in SSA				
Central Africa		1	1	1
East Africa			1.13 (1.09, 1.16)	1.08 (1.04, 1.13)*
Southern Africa			1.15 (1.08, 1.23)	1.06 (0.97, 1.16)
West Africa			1.10 (1.07, 1.13)	1.04 (0.99, 1.08)
Country income level in SSA				
Lower		1	1	1
Lower-middle			0.96 (0.94, 0.98)	0.95 (0.92, 0.98)*
Upper-middle			1.00 (0.94, 1.07)	1.01 (0.93, 1.10)
ICC (%)	14.9 (14.0,15.8)	13.0 (11.5, 14.6)	14.3 (13.4, 15.3)	13.2 (11.6, 14.9)
MOR	2.38			
Log-Likelihood	−46249.74	−21045.82	−42763.05	−18752.76
Deviance	92499.48	42091.64	85526.1	36505.52
AIC	92501.48	42129.63	85542.11	37557.51
BIC	92510.32	42282.96	85612.21	37764.28

*p-value < 0.05.

## Discussion

This study attempted to determine the pooled prevalence and factors associated with healthcare-seeking behavior for diarrhea in children under the age of 5 years. The pooled prevalence of HSB for diarrhea in under-five children in SSA was 58.71%, which is lower than that reported in studies performed in Bangladesh (19) and Indonesia (37). Our findings also revealed that the mother’s age, maternal educational status, the mother’s working status, ANC visits, postnatal checkups for the baby, wasting, distance to the health facility, SSA region, and the country’s income level were significant factors associated with healthcare-seeking behavior for diarrhea in children under the age of 5 years.

Mothers aged 20–35 years were more likely to engage in healthcare-seeking behavior for their children with diarrhea than mothers aged 15–19 years. This finding is similar to those of previous studies (38, 39). The possible reason could be that older mothers may have greater financial means and decision-making power, allowing them to prioritize their children’s health and make healthier choices than younger mothers do (38).

The prevalence of seeking healthcare for children under the age of 5 years with diarrhea was greater among working mothers than among non-working mothers. This finding is similar to those of previous studies (40). The possible reason could be that mothers who work can afford to pay for healthcare services for their children when they are sick (41, 42). The other possible reason might be that employed mothers have more autonomy in making healthcare decisions (41, 43).

Mothers who attained primary, secondary, or higher education have more healthcare-seeking behavior for their children with diarrhea than mothers with no formal education. The possible reason could be that mothers who are more educated often have higher incomes, allowing them to afford appropriate healthcare for their children (44, 45). The other possible justification could be that educated mothers are more knowledgeable about diseases and their consequences, which will improve their health-seeking behavior (38).

Maternal ANC visit was found to be a significant predictor of HSB for diarrhea among under-five children. This finding is consistent with previous evidence (46). The possible reason might be that women who have ANC visits may have an opportunity for health education, which increases the likelihood of mothers seeking healthcare for their children (47, 48).

Under-five children who were moderately or severely wasted had a greater prevalence of receiving healthcare for diarrhea than normal children. Previous studies have also shown that mothers who have children with more than one symptom have better healthcare-seeking behavior (49). Under-five children who had a postnatal checkup within 2 months of delivery received increased healthcare for diarrhea compared to those who did not have a postnatal checkup. This finding is similar to those of previous studies (40, 46). A possible reason might be that mothers who engage in postnatal checkups receive valuable medical advice, which positively influences their healthcare-seeking behavior (50).

The prevalence of seeking healthcare for children with diarrhea was greater among those who had no big problem with distance to health facilities. This finding is similar to those of previous studies (37, 51, 52). The possible reason might be that when the distance to the health facility is not a large concern, it will not impose any hurdles because there are reduced direct and indirect costs of seeking healthcare, such as fees for transportation and being off from work.

Children under the age of 5 years in lower-middle-income SSA countries were less likely to receive healthcare for diarrhea than those from lower-income SSA countries. In addition, children from East Africa were more likely to receive healthcare for diarrhea than those from Central Africa. This may be explained by socio-economic and socio-cultural differences and disparities in healthcare accessibility across regions.

### Strengths and limitations of the study

This research is a multicountry study that utilized the most recent DHS data from 35 SSA countries, incorporating a substantial sample size. To ensure accurate and reliable estimations, the data were appropriately weighted. To account for clustering effects and improve the accuracy of our standard errors and estimates, we used multilevel modeling. In addition, to address the issue of overestimation of effect size often seen in traditional multilevel binary logistic regression models used in cross-sectional studies, we employed multilevel robust Poisson regression, which made our findings more precise (33). Despite the strengths mentioned above, the cross-sectional nature of this study precluded us from establishing a cause–effect relationship. Mothers provided all the information about diarrhea and HSB, which could introduce recall bias and subjectivity. In addition, by merging DHS data from different countries, important contextual differences between countries might be overlooked, which may influence the variables being measured. As DHS data for each nation were not collected simultaneously, changes may go unnoticed, leading to biased comparisons.

## Conclusion

According to our study, the proportion of under-five children with diarrhea receiving healthcare in SSA was lower than that in developed countries. The mother’s age, maternal educational status, the mother’s working status, ANC visits, postnatal checkups for the baby, wasting, distance to the health facility, SSA region, and the country’s income level were found to be significant factors associated with HSB for diarrhea in children under the age of 5 years. To improve healthcare-seeking behavior, policymakers and stakeholders should enhance public health interventions by considering the factors associated with healthcare-seeking behavior for children under the age of 5 years with diarrhea. This is particularly important in SSA countries with a low prevalence of such behavior. Effective health policy interventions, such as improving the education and employment status of mothers, promoting antenatal and postnatal care visits, building health facilities in proximity, and raising awareness among mothers of malnourished children to seek healthcare services, are necessary to enhance healthcare-seeking behavior.

## Data Availability

Data is publicly available and can be accessed from: https://dhsprogram.com/data/dataset_admin/login_main.cfm?CFID=10818526&CFTOKEN=c131014a480fe56-4E0C6B7F-F551-E6B2-50.
